# The Clinical Characteristics and Antimicrobial Resistance of *Staphylococcus aureus* Isolated from Patients with Staphylococcal Scalded Skin Syndrome (SSSS) in Southwestern China

**DOI:** 10.3390/antibiotics13060516

**Published:** 2024-05-31

**Authors:** Yidan Wu, Hengfeng Wu, Man Wu, Wanchen Wei, Yuying Wei, Tiantian Li, Cunwei Cao, Zhijian Yao

**Affiliations:** 1 Department of Dermatology and Venereology, The First Affiliated Hospital of Guangxi Medical University, Nanning 530021, China; dandan_513@163.com (Y.W.); mandylttgxmu@163.com (T.L.); 2 Guangxi Key Laboratory of Mycosis Prevention and Treatment, Nanning 530021, China; 3 Department of Clinical Medicine, Guangxi Medical University, Nanning 530021, China; m15977516899@163.com (H.W.); wuumman@163.com (M.W.); 18378769553@163.com (W.W.); weiyuying141@163.com (Y.W.); 4 Guangxi Scientific and Technological Innovation Cooperation Base of Mycosis Prevention and Control, Nanning 530021, China

**Keywords:** staphylococcal scalded skin syndrome (SSSS), bacterial infectious dermatosis, methicillin-resistant *Staphylococcus aureus* (MRSA), retrospective study, antimicrobial susceptibility

## Abstract

Staphylococcal scalded skin syndrome (SSSS) is a rare, toxin-mediated, desquamating bacterial infectious dermatosis. So far, data from Southwestern China is scarce. This study aimed to investigate the clinical characteristics of SSSS patients in our hospital, the relative proportion of methicillin-resistant *Staphylococcus aureus* (MRSA) in skin and soft tissue secretions, and the drug sensitivity of *S. aureus* to better assist dermatologists in the diagnosis and treatment of SSSS. We reviewed the demographic characteristics, clinical manifestations, treatment regimens, therapeutic efficacy, laboratory test results, drug sensitivity, and outcome data of 79 SSSS patients from January 2012 to December 2021. Statistical analysis was performed using *t* tests and chi-square tests. Among the 79 SSSS patients, MRSA was detected in 35 (44.3%) isolates: 34 community-acquired (CA)-MRSA (97.1%) and 1 hospital-acquired (HA)-MRSA. The SSSS incidence increased annually from 2012 to 2014 and then decreased gradually after peaking in 2015. All the isolates were sensitive to vancomycin, tigecycline, linezolid, moxifloxacin, levofloxacin, and ciprofloxacin; were completely resistant to penicillin; and had low sensitivity to clindamycin and erythromycin. Interestingly, the sensitivity of MRSA to tetracycline increased annually after 2015. The resistance rates to common drugs previously used to treat SSSS increased. These findings may accelerate diagnosis and improve empirical antibiotic use, suggesting that clinicians should prescribe drugs according to antimicrobial susceptibility.

## 1. Introduction

Staphylococcal-scalded skin syndrome (SSSS) is a potentially life-threatening disorder that is considered a pediatric emergency. It is a superficial blistering skin disease caused by exfoliative toxins released by *Staphylococcus aureus* (*S. aureus*) [1]. It mainly occurs in infants ≤5 years of age, but a small number of cases in which the disease occurs in adults with suppressed immunity or some underlying disease have been described. The risk factors for SSSS include renal insufficiency, immunodeficiency, and inadequate prevention and control of nosocomial infections [2]. Previous epidemiological investigations revealed that most SSSS cases were caused by methicillin-sensitive *S. aureus* (MSSA) [3], but in recent years, a few authors have reported SSSS cases related to methicillin-resistant *S. aureus* (MRSA) [4].

SSSS is a potentially fatal condition characterized by skin erythema and superficial blisters caused by the release of epidermal exfoliation toxins by *S. aureus*. SSSS often starts with superficial blisters and skin erythema, but the exfoliative toxins produced by bacteria can cause large areas of skin exfoliation, which can lead to sepsis, pneumonia, dehydration, electrolyte disorders, and other severe complications [2]. Epidemiological studies conducted in multiple countries, including France [5], Germany [6], the Czech Republic [7], and the United States [3], have separately reported incidence rates of SSSS of 0.09–0.13, 0.56, 251.1 cases per million, and 7.67 cases per million, respectively. A recent study in China [8] revealed a notably higher incidence of SSSS, with 696.4 cases per million people in the studied area. Timely detection and treatment of SSSS are crucial because the condition can rapidly deteriorate, potentially leading to patient mortality. In recent years, cases of SSSS infection due to MRSA have been increasingly reported in China and other countries. MRSA-infected patients usually have longer treatment durations, higher costs, and poorer prognoses than MSSA-infected patients. Therefore, it is imperative to improve the early detection and diagnosis of SSSS, proactively prevent MRSA infections, and focus on prevention and monitoring strategies for patients who have been infected with MRSA.

*S. aureus* is a Gram-positive bacterium that readily causes clinical infections and colonizes human skin and mucosal surfaces. It is a facultative anaerobic bacterium that is adaptable to a variety of environments and is ubiquitous in the air and some polluted environments [9]. *S. aureus* is also a common pathogen in children and adolescents and can cause serious localized or systemic infections, bacteremia, infective endocarditis, and bone, joint, skin, and soft tissue infections [10]. According to the sensitivity of *S. aureus* strains to methicillin, they used to be delineated as MRSA and MSSA. MRSA isolates were first detected in a hospital environment and were soon reported in the community [11]. According to epidemiological characteristics and virulence, MRSA isolates are divided into community-acquired MRSA (CA-MRSA) and hospital-acquired MRSA (HA-MRSA) [12]. According to epidemiological investigations of SSSS in different countries, the incidence of this disease has been low in recent years. However, SSSS is still an important condition because it can endanger life at any time [3]. There are few reliable or large-sample studies on the different subtypes of *S. aureus,* or SSSS, caused by CA-MRSA and HA-MRSA. Moreover, in a previous study on the clinical distribution and disease type of MRSA infections in our hospital, we found that SSSS was one of the primary outcomes of CA-MRSA infection. SSSS may cause a poor prognosis, a longer length of stay, and a greater economic burden. Therefore, it is necessary to improve the corresponding diagnosis and treatment and to develop preventive measures. For further clinical analysis, in this study, we retrospectively analyzed data on SSSS infection, clinical characteristics, and changes in drug resistance in our hospital over ten years, from January 2012 to December 2021. Moreover, we meticulously classified the isolated *S. aureus* groups using oxacillin or cefoxitin and a VITEK-2 Compact automatic microbiological analyzer. We determined the minimum inhibitory concentrations (MICs) of the strains by the broth microdilution method. The clinical data were comprehensively interpreted to provide a solid foundation for the clinical diagnosis, treatment, and prevention of SSSS. Our findings revealed that nearly half of the patients in our study had SSSS caused by CA-MRSA, and these cases showed increased resistance to common drugs previously used to treat SSSS.

According to previous research by our group, the most common skin disease caused by a CA-MRSA infection is SSSS [13]. At present, there is a lack of data on the epidemiological and clinical characteristics of SSSS patients infected with MRSA isolates in Southwestern China. Our study contributes to the understanding of the clinical characteristics, bacterial species distribution, and antimicrobial susceptibility of MRSA-infected SSSS patients in Southwestern China, laying the groundwork for future investigations.

## 2. Results

### 2.1. Demographic and Clinical Characteristics of Patients with SSSS

**Figure 1:**
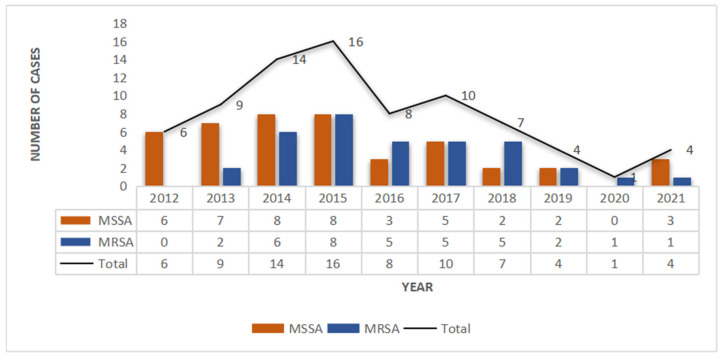
Methicillin-resistant *S. aureus* (MRSA) and methicillin-susceptible *S. aureus* (MSSA) infections in SSSS patients from 2012 to 2021.

A total of 79 SSSS cases were analyzed from January 2012 to December 2021. Among them, the number of SSSS cases diagnosed from 2014 to 2017 was relatively high, and the overall incidence rate showed a decreasing trend (Figure 1). According to the seasonal distribution analysis, the greatest percentage of patients (*n* = 32, 47%) were identified in October to December. Out of 291 patients clinically diagnosed with SSSS, we screened those who had not received postadmission antibiotics and whose skin and soft tissue secretion samples were collected within 24 h of admission. Patients with negative secretion cultures were excluded, resulting in 79 eligible patients. Among the 79 patients in this study, the youngest was 1 year old, the oldest was 19 years old, and the average age was 3.41 years (3.41 ± 3.01). Moreover, there were 50 males (63.3%) and 29 females (36.7%), and there was no significant difference in the distribution of MRSA and MSSA by sex. The shortest length of hospital stay was 4 days, the longest was 21 days, and the average length of hospital stay was 6.93 days (6.93 ± 2.68). By the end of the study period, 55 patients were cured (69.6%), 24 improved (30.4%), and 0 died. Twenty-three patients had complications other than SSSS, including dermatoses, such as atopic dermatitis, pustulosis, and drug-induced dermatitis; genetic diseases, such as G6PD deficiency; infectious diseases, such as *Mycoplasma pneumoniae* infection; acute tonsillitis; burns; scalp contusions; dermatectomy; debridement; and other operations. Most of the patients were of Han or Zhuang nationality (Table 1).

Twenty percent of the patients had fever before admission, and 70% of the patients had fever during hospitalization. The lesions were mainly located in the head/face/neck (96.2%), followed by the torso (77.2%). There were no lesions in the oral mucosa of any of the patients with SSSS. Laboratory examination results indicated that the white blood cell (WBC) count of patients with SSSS was greater than normal, and the CRP level was greater in patients with MRSA infection. Moreover, there was no significant difference in any laboratory test result between patients with MSSA infections and patients with MRSA infections.

### 2.2. Therapeutic Regimen of Patients with SSSS

**Figure 2:**
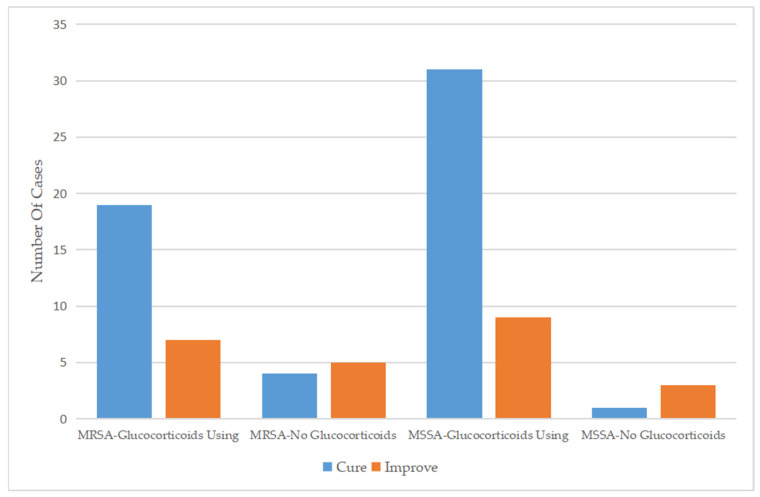
Comparison of the efficacy of glucocorticoid use between 2012 and 2021.

Regarding the therapeutic regimen and treatment course, the duration of antibiotic use for patients infected with MSSA was 6.59 ± 2.81 days, which was similar to the 6.66 ± 2.07 days for patients infected with MRSA. Almost all patients were treated with a cephalosporin, mainly third-generation cephalosporins, as monotherapy. In addition, a small number of patients were treated with cephalosporin combined with other types of antibiotics or other types of antibiotics alone. Approximately 30% of the patients had been treated with antibiotics before admission, while only 14 (17.7%) patients continued to use antibiotics after discharge (Table 2). For patients who used drugs that affect immune function, glucocorticoids were more commonly used in patients infected with MSSA (90.9%) than in those infected with MRSA (74.3%) (Figure 2), while only five patients were treated with gamma globulin (Table 3). Glucocorticoids combined with antibiotics did not significantly affect the length of hospital stay among patients infected with MRSA or MSSA but did significantly improve clinical outcomes.

### 2.3. Differences in MRSA and MSSA Infections among SSSS Patients

From January 2012 to December 2021, 79 culture-positive cases were selected from 291 SSSS patients, 35 (44.3%) of which were caused by MRSA, including 34 CA-MRSA strains and 1 HA-MRSA strain, and 37 (55.7%) of which were caused by MSSA. Thus, in the past 10 years, the average detection rate of MRSA isolated from the skin and soft tissue secretions of SSSS patients was 44.3%, accounting for nearly half of all *S. aureus* infection cases, with CA-MRSA accounting for 97.1% (30/31) and HA-MRSA accounting for 2.9% (1/31). Over the past 10 years, the number of MRSA isolates increased gradually in the first four years and then decreased gradually after reaching a peak in 2015 (*p* = 0.026), while the number of MSSA isolates decreased annually (Figure 1).

### 2.4. Antimicrobial Susceptibility of S. aureus Clinical Isolates

**Figure 3:**
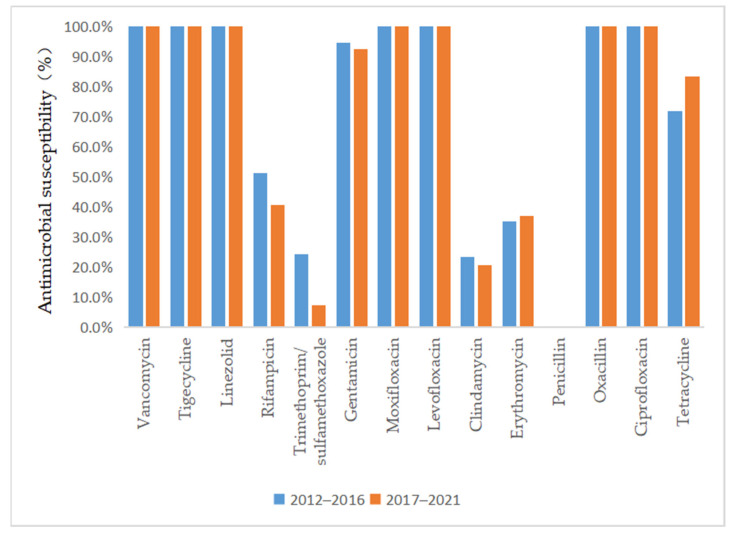
Comparison of the antimicrobial susceptibility of isolates from patients with MSSA infection between 2012–2016 and 2017–2021.

**Figure 4:**
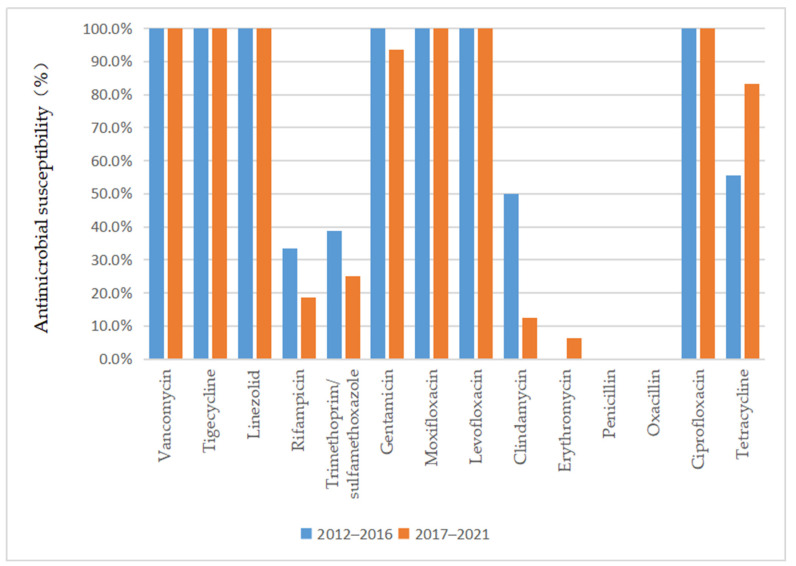
Comparison of the antimicrobial susceptibility of isolates from patients with MRSA infection between 2012–2016 and 2017–2021.

As depicted in Figure 3 and Figure 4, vancomycin, tigecycline, linezolid, moxifloxacin, levofloxacin, and ciprofloxacin were effective antibiotics against MRSA and MSSA, with a 100% susceptibility rate. In addition, MRSA maintained a high susceptibility to teicoplanin, daptomycin, and gentamicin. Complete resistance to penicillin G and oxacillin was detected in MRSA isolates during the 10-year study period. The susceptibility rates of MRSA to clindamycin, erythromycin, sulfamethoxazole, and rifampicin were also low. However, the sensitivity of MRSA to tetracycline increased annually after 2015 (*p* = 0.02) and reached 100% by 2018. MSSA showed a high susceptibility to gentamicin and had antibiotic resistance to penicillin G. Furthermore, we found that the resistance rate to rifampicin and trimethoprim/sulfamethoxazole decreased, while that to clindamycin, erythromycin, and tetracycline increased in MSSA. 

## 3. Discussion

This study aimed to describe the relative proportion of MRSA, etiology, clinical features, and drug susceptibility of *S. aureus* in the skin and soft tissue secretions of patients with SSSS to aid dermatologists in the diagnosis and treatment of SSSS. To our knowledge, there has rarely been a large-scale, long-term SSSS cohort with MRSA infections in Southwestern China. This study investigated the distribution and antibiotic susceptibility of MSSA and MRSA strains isolated from skin and soft tissues. Our results showed that MRSA infection accounted for nearly half of all SSSS patients, and almost all infections were CA-MRSA, which is consistent with our previous research. All the isolates were sensitive to vancomycin, tigecycline, linezolid, moxifloxacin, levofloxacin, and ciprofloxacin and were generally resistant to penicillin, clindamycin, and erythromycin. In addition, we analyzed the demographics, clinical characteristics, and treatment options of patients with culture-positive isolates and found that the skin lesions of all patients did not invade the oral mucosa. In addition, cephalosporins were the most commonly used therapeutic agents in this study, and glucocorticoids were widely used in combination with antibiotics to treat SSSS. These data can help clinical dermatologists understand the etiology, clinical features, and drug susceptibility of this disease to improve antibiotic treatment.

Accordingly, this study included a comprehensive analysis of the clinical characteristics, therapeutic regimens, curative outcomes, proportion of patients with MRSA infection, and drug resistance of SSSS patients in our hospital, which provides a reference and important insight for the diagnosis and treatment of SSSS. In addition, there are a few retrospective studies, including clinical data analysis, strain typing, or drug sensitivity analysis of SSSS patients in Southwestern China. This study provides information and a reference for diagnosis and treatment in this field.

According to previous studies, China has a high incidence of SSSS [8]. Notably, the overall incidence of SSSS showed a downward trend. This might be due to the improvement of diagnosis and treatment by dermatologists in municipal and county hospitals with the strengthening of medical technical training and continuous education. As a result, there were fewer patients in our Grade A tertiary referral hospitals. In this study, SSSS often occurred from October to December, which is not consistent with the conclusions of other studies showing a peak incidence in the summer and fall [2]. We speculate that it might be related to the unique climate of Southwest China, such as temperature and humidity. The reason is worthy of further exploration. SSSS was more common in males than in females [8], with an average age less than 3 years and an average LOS of approximately 7 days [14], which is consistent with the findings of other studies in China. Most of the patients recovered and were discharged, and the factors influencing outcomes might be related to the simultaneous existence of other skin diseases, genetic diseases, trauma, surgery, and other factors affecting healing time. Most of the SSSS patients were of Han or Zhuang nationality, which was consistent with the ethnic composition of this area. The lesions were mainly located in the head/face/neck (96.2%), followed by the torso (77.2%). In the laboratory examinations, the WBC count and the CRP value were both increased in SSSS patients. These results were consistent with the pathogenesis of SSSS and might reveal that there was not much difference between MSSA and MRSA infections in blood.

In order to better distinguish from other diseases, we also made an in-depth analysis of the location of SSSS. Because of their similar clinical manifestations and skin lesions, it is difficult to distinguish SSSS from Stevens–Johnson syndrome and toxic epidermal necrolysis (TEN). Previous studies have shown that differentiation among SSSS, Stevens–Johnson syndrome, and TEN mainly depends on skin biopsy; however, skin biopsy is invasive and takes a long time to obtain results, which impacts the time until clinical diagnosis and treatment [15]. In this study, we found that the skin lesions of all SSSS patients mainly appeared around the mouth and did not invade the oral mucosa, which was consistent with the findings of a previous study showing that the mucosa of SSSS patients was not affected [16,17]. For Stevens–Johnson syndrome and TEN, the vast majority of patients have mucosal involvement, which typically manifests as full lip surface crusting, denudation, and pain [18,19,20]. This difference in clinical manifestations suggests that oral mucosal involvement might be a simple method for the early, noninvasive, and timely differential diagnosis of SSSS and TEN when a clinical pathological biopsy has not been confirmed. Therefore, we speculated that observing whether the oral mucosa was damaged might be a key point in distinguishing SSSS from severe drug eruptions, which is helpful for clinicians to make an early diagnosis of the disease at admission and is highly important for the timely and reasonable treatment of patients. Many studies are needed to confirm this theory. In summary, our findings mirrored those of previous studies. We noted the absence of oral mucosal involvement and that the oral mucosa was not invaded in any of the patients, potentially serving as a crucial distinguishing factor from other similar conditions.

In addition to analyzing clinical symptoms, we investigated treatment options and duration. Regarding the therapeutic regimen and course of treatment, the duration of antibiotic treatment in patients infected with MSSA was 6.59 ± 2.81 days, which was not significantly different from that in patients infected with MRSA. Almost all patients were treated with cephalosporins plus glucocorticoids, mainly with third-generation cephalosporins, including ceftriaxone, cefotaxime, cefixime, and cefaclor. Only a few patients were treated with cephalosporins combined with other types of antibiotics or with other types of antibiotics alone. The three patients treated with cephalosporins combined with aminoglycoside antibiotics, such as vancomycin and teicoplanin, were all SSSS patients with MRSA infection, and after treatment, all three patients were discharged from the hospital. Previous reports have noted that cephalosporins combined with aminoglycoside antibiotics could yield better curative effects for some patients with severe MRSA infections [21]. The use of glucocorticoids was more common in patients infected with MSSA (90.9%) than in those infected with MRSA (74.3%). In our study, for almost all patients treated with glucocorticoids plus antibiotics, considerable efficacy of the treatment was observed. Patients infected with MRSA (*p* < 0.001) or MSSA (*p* = 0.024) presented significant improvement in symptoms upon discharge. However, whether glucocorticoids were used had no significant effect on the length of the hospital stay. Previous studies outside China have shown that the use of corticosteroids, especially systemic steroids, should be prohibited because these drugs can reduce immune function and aggravate the disease [1,20]. However, a Chinese study reported that antibiotics combined with glucocorticoids did not result in significant side effects due to the inclusion of glucocorticoids [14]. We hypothesized that glucocorticoids were used in SSSS treatment to counter the release of epidermal exfoliation toxins, which has a similar effect to that of glucocorticoid treatment in sepsis because both act to modulate the immune response to infection [22]. Glucocorticoids and antibiotic therapy have been shown to be beneficial for patients. In our research, only 5 SSSS patients were treated with antibiotics combined with gamma globulin; one patient was infected with MSSA, and the other four patients were infected with MRSA. Moreover, the patient with MSSA infection achieved improvement and was discharged from the hospital, while the four patients with MRSA infection were completely cured. A systematic review concluded that intravenous immunoglobulin was recommended for the treatment of SSSS [15]. However, a recent study linked intravenous immunoglobulin use to extended hospitalization [2]. In addition, most SSSS patients were children, and early diagnosis and standardized treatment are very important in this demographic group. In our study, SSSS patients infected with MRSA accounted for nearly half of all patients, and public health problems should receive more attention from relevant departments. In terms of treatment options, combining glucocorticoids with antibiotics was effective, challenging the belief that glucocorticoids are contraindicated.

The detection rate of MRSA in SSSS patients in this study was 44.3%, which was different from the predominance of MSSA isolates in Europe and the United States [5,23,24]. However, individual reports in Korea [4] and Taiwan [25] also showed that MRSA isolates accounted for a large proportion of patients. CA-MRSA was dominant among the MRSA infections, suggesting that the MRSA infections of the SSSS patients in this study mainly originated from the community and that it is necessary to strengthen the prevention and control of community infections. Moreover, detection, centralized management, and treatment should be emphasized and implemented as early as possible. In addition, from 2012 to 2021, the incidence of MRSA and MSSA infections in SSSS patients mainly showed a downward trend, which was consistent with the incidence of SSSS during this period. Notably, a significant decline was observed in 2020 and 2021, which might be related to the strict prevention and control measures taken during the COVID-19 pandemic. On the one hand, more stringent infection prevention and control measures resulted in a reduction in the number of outpatient visits. On the other hand, with the improvement of medical and diagnostic technology in primary hospitals, the diagnosis and treatment of SSSS improved, so the number of patients referred to our hospital, which is a tertiary care center, from other hospitals at all levels decreased, which might also be one of the reasons for the sharp decline in the number of patients.

Regarding the antimicrobial susceptibility of *S. aureus* clinical isolates from SSSS patients, we observed that a significant proportion of the MRSA isolates were susceptible to vancomycin, teicoplanin, linezolid, and other potent antibiotics. The isolates also had a high rate of susceptibility to quinolone antibiotics, while they were mainly resistant to clindamycin, which is commonly used in the clinic. This finding demonstrated that better clinical outcomes can be expected if empirical antibiotic treatment is followed by early adjustment to the use of antibiotics to which the isolate was sensitive according to susceptibility testing. Interestingly, our study showed that the sensitivity of MRSA to tetracycline increased annually, which might be related to the lack of clinical tetracycline use. However, previous studies have shown that tetracycline is unsuitable for oral use in children under 8 years of age because of its effect on tooth and bone development [26]. Although the SSSS patient group comprised mainly children, whether tetracyclines could be used to treat SSSS patients infected with MRSA remains to be verified by clinical application. Patients infected with MSSA were not only sensitive to vancomycin, teicoplanin, and linezolid but also highly sensitive to quinolones, cephalosporins, and aminoglycosides, which could be considered in clinical treatment. However, although these isolates were highly sensitive to quinolones, quinolones are not suitable for clinical application because of their side effects in children. Similar to MRSA, MSSA was also resistant to clindamycin and erythromycin, which might be due to the overuse of these antibiotics in previous years. In addition, the sensitivity to rifampicin and trimethoprim/sulfamethoxazole increased annually, which could be associated with their lower use in the clinical treatment of this disease. Nevertheless, we recommend that clinicians choose antibiotics based on the results of the latest antibiotic profile. This approach can not only provide a better curative effect but also reduce the development of antibiotic resistance.

Our study had several limitations. This study was conducted at one university hospital; therefore, the data do not represent provincial SSSS epidemiology. Because this was a retrospective study, we were unable to determine the genotypes of *S. aureus*, so we could distinguish whether the isolate was derived from the community or hospital only through epidemiology. Furthermore, some specific factors, including other *S. aureus* colonization conditions, hospitalization history, and treatment efficacy at discharge, were difficult to trace. In addition, it was difficult to determine whether *S. aureus* isolates were the actual cause of the disease. Furthermore, the diagnosis of some complications might have occurred later than those of SSSS, although those diagnoses were listed in the discharge diagnosis together with SSSS. Therefore, associations that might affect the cure rate need to be further confirmed. Finally, our research was carried out in a tertiary care center, which provided high-level specialist services, diagnosed and initiated treatment for critical and difficult diseases, and performed two-way referrals. Thus, the investigation results might not clearly reflect problems in the community. In summary, additional prospective and multicenter studies are needed to accurately classify the strains isolated from SSSS patients, collect complete clinical data, evaluate the treatment effect more rigorously, further explore the distribution of MSSA and MRSA, and generalize treatment outcomes across the entire region.

## 4. Materials and Methods

### 4.1. Patient Identification and Medical Chart Review

A retrospective analysis of 291 patients with SSSS treated at our hospital from 1 January 2012, to 31 December 2021, was performed using the hospital information system (HIS). All newly admitted patients without prior antibiotic use whose skin and soft tissue secretion samples were collected within 24 h of admission were selected for further investigations. Patients with negative secretion cultures were excluded. Seventy-nine strains of *S. aureus* were successfully isolated and cultured, including 35 MRSA isolates and 44 MSSA isolates. The exclusion criteria were as follows: (1) repeated results from the same patient, with the first samples with a positive culture retained; (2) samples that were possibly contaminated and not secreted by skin and soft tissues, such as urine and blood; (3) samples that were not submitted for examination in a timely manner (within 2 h); and (4) data from patients with incomplete medical records. We defined HA-MRSA and CA-MRSA infections according to the recommendations of the CDC and Naimi et al. [27,28]. HA-MRSA patients were defined as patients who (1) had a MRSA infection detected 48 h after admission; (2) had a history of hospitalization, surgery, dialysis, or long-term care facility residence within 1 year from the date of MRSA culture; (3) had a permanent presence of MRSA infection at the time of culture with an indwelling catheter or percutaneous medical device; or (4) had previous positive MRSA culture results during the study period. All other patients were classified as having CA-MRSA.

We collected information on the patients’ demographics (including sex, age, nationality, and season of hospitalization), clinical manifestations (including prehospitalization fever, complications, previous trauma, previous surgery, and the presence of a foreign body in the lesion), course of hospitalization (including length of stay, presence and duration of fever during hospitalization, course of treatment with antibiotics, and use of drugs affecting immune function), and therapeutic outcomes.

**Figure 5:**
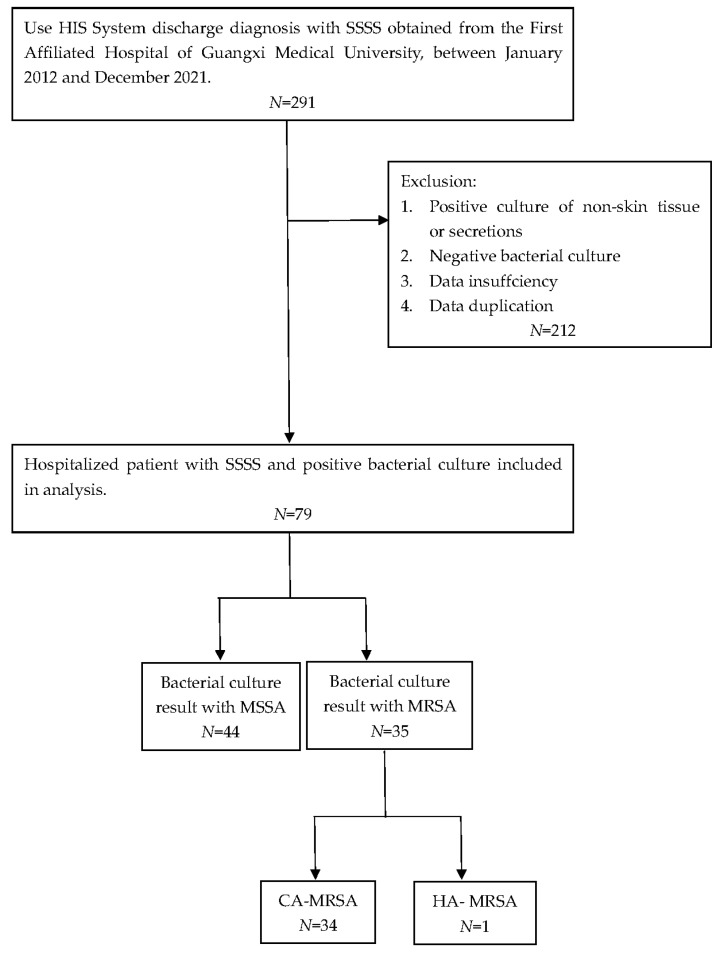
Flow chart of the exclusion and inclusion criteria for this study.

In addition, laboratory evaluations at presentation (including white blood cell count, hemoglobin level, platelet count, erythrocyte sedimentation rate (ESR), and C-reactive protein (CRP) level) and antimicrobial susceptibility test results were reviewed (Figure 5).

### 4.2. Strain Identification and Drug Sensitivity Analysis

We distinguished MSSA from MRSA by using an oxacillin (MIC > 4 μg/mL) or cefoxitin test (positive screening result) and a VITEK-2 Compact automatic microbiological analyzer [29]. Isolates resistant to oxacillin (MIC > 4 µg/mL) or cefoxitin (>32 µg/mL) were classified as MRSA. The VITEK-2 Compact automatic microbiological analysis and identification instrument were obtained from the French Biological Merier Company (Villetaneuse, France), as was the identification card. The quality control strain was *S. aureus* ATCC25923, which was provided by the temporary inspection center of the Ministry of Health. Moreover, the susceptibility cards of the instrument outlined above were used to determine the MICs of the strains via the broth microdilution method. Some of the supplementary drug sensitivity test paper was purchased from Oxoid Company, Basingstoke, the United Kingdom, and the drug susceptibility test was performed by the paper diffusion method (K-B method). Mueller-Hinton (MH) medium from Zhengzhou Antu Bioengineering Co., Ltd. (Zhengzhou, China) was used for the drug susceptibility tests. The final interpretation and susceptibility results were determined based on the CLSIM100 standard [30].

### 4.3. Statistical Analysis

SPSS 25.0 software was used for statistical analysis. The measurement data are expressed as X ± S and were compared by the *t* test, while the count data are expressed as n%, and the X2 test was used for comparison. For analysis, we divided the data into two five-year intervals: January 2012 to December 2016 (the first five years) and January 2017 to December 2021 (the last five years). All analyses were two-tailed, and *p* < 0.05 was considered to indicate statistical significance.

## 5. Conclusions

In summary, this study provides a summary of the clinical characteristics, distribution, and drug sensitivity of MRSA and MSSA infections in patients with SSSS in the study area over the past 10 years. Our exploration of the sites, complications, treatment outcomes, and therapy of skin lesions will help improve clinician awareness of this disease as well as the therapeutic effects. In addition, to help address the lack of epidemiological investigations related to SSSS, the proportion of drug-resistant bacteria, and drug sensitivity in Southwestern China, this study provides long-term and reliable data. This information could help clinicians better understand the current epidemiological characteristics of SSSS patients and the drug sensitivity of isolates to reduce the misdiagnosis rate, provide evidence-based empirical treatment, reduce the number of hospitalization days, and reduce patients’ economic burden and psychosocial stress. Notably, hospitals should strengthen their prevention and control measures for drug-resistant bacteria to address the relatively large proportion of drug-resistant infections. Overall, strictly managing the disinfection and isolation of related patients and maintaining the declining trend in the incidence of drug-resistant bacterial infections each year remain important.

## Figures and Tables

**Table 1:** Characteristics of hospitalized children with SSSS from 2012 to 2021.

Characteristic	MSSA (*N* = 44)	MRSA (*N* = 35)	*p* Value
Demographic			
Female	12 (27.3%)	17 (48.6%)	0.056
Age	3.68 ± 3.54	3.05 ± 2.18	0.338
Ethnicity			0.180
Han nationality	18 (40.9%)	18 (51.4%)	
Zhuang nationality	23 (52.3%)	17 (48.6%)	
Other ethnic group	3 (6.8%)	0	
Month ^a^			0.469
January to March	9 (20.4%)	2 (5.7%)	
April to June	5 (11.4%)	7 (20%)	
July to September	11 (25.0%)	12 (34.3%)	
October to December	19 (43.2%)	14 (40%)	
Clinical presentation			
Prehospitalization fever	9 (20.4%)	6 (17.1%)	
Fever days during hospitalization	1.57 ± 1.76	1.29 ± 1.23	0.422
Fever during hospitalization	31 (70.4%)	25 (71.4%)	
Length of stay	7.09 ± 3.29	6.74 ± 1.65	0.570
Therapeutic effect evaluation			0.507
Cure	32 (72.7%)	23 (65.7%)	
Improvement	12 (27.3%)	12 (34.3%)	
Location ^b^			0.843
Extremities	29 (65.9%)	22 (62.9%)	<0.001 *
Head/Face/Neck	42 (95.4%)	34 (97.1%)	
Groin/Perineum/External genitalia	19 (43.2%)	16 (45.7%)	<0.001 *
Fossa axillaris	16 (36.4%)	17 (48.6%)	<0.001 *
Buttock/Perianal region	7 (15.9%)	4 (11.4%)	<0.001 *
Torso	38 (86.3%)	23 (65.7%)	<0.001 *
Laboratory data at presentation			
WBC count (10^9^/L)	13.52 ± 6.01	12.15 ± 4.85	0.277
Hb (g/L)	117.91 ± 20.7	122.12 ± 11.72	0.287
Platelet count (10^9^/mL)	408.99 ± 118.61	392.41 ± 114.11	0.532
CRP (mg/dL)	7.83 ± 10.81	8.81 ± 12.59	0.749
ESR (mm)	10.41 ± 7.42	8.5 ± 4.52	0.281
Comorbidities ^c^			0.605
None	29 (65.9%)	27 (77.1%)	
Dermatologic disease	9 (20.4%)	2 (5.7%)	
Genetic syndrome/Metabolic disease	4 (9.1%)	3 (8.6%)	
Infectious disease	7 (15.9%)	2 (5.7%)	
Trauma	2 (4.5%)	0	
Surgery	3 (6.8%)	1 (2.9%)	

^a^ Month: We divide the year into 4 parts:January to March, April to June, July to September, and October to December. The average temperature (°C) corresponding to each monthly interval in the 10 years from 2012 to 2021 is as follows: 15.88, 26.10, 27.99, and 19.19. The average precipitation (mm): 58.42, 145.09, 186.15, and 72.82. The relative humidity (%): 79.18%, 79.12%, 78.77%, and 73.82%. (All meteorological data were derived from: https://www.wheata.cn/, accessed on 7 May 2024). ^b^ Location: the location of lesions on the body. There was no significant difference between MSSA and MRSA isolation in each location. For the head/face/neck where lesions occurred most frequently, there was a significant difference in the occurrence of lesions in other parts of the body (* *p* < 0.001). ^c^ Comorbidities: Dermatologic disease: atopic dermatitis, pustulosis, drug dermatitis. Genetic syndrome/metabolic disease: G6PD deficiency. Infectious diseases: Mycoplasma pneumoniae infection, acute tonsillitis. Trauma: burns, scalp contusions. Surgery: excision of the skin lesion and debridement. Abbreviations: LOS, length of stay; WBC, white blood cell count; CRP, C-reactive protein; Hb, hemoglobulin; MSSA, methicillin-susceptible *S. aureus*; MRSA, methicillin-resistant *S. aureus*; SSSS, staphylococcal scalded skin syndrome. * Denotes a significant difference between methicillin-resistant *S. aureus* and methicillin-susceptible *S. aureus* at *p* < 0.05.

**Table 2:** Treatment of hospitalized children with SSSS from 2012 to 2021.

Characteristic	MSSA (*N* = 44)	MRSA (*N* = 35)	*p* Value
DOTs (intravenous + oral)	6.59 ± 2.81	6.66 ± 2.07	0.908
Empiric antibiotic ^d^			
Cephalosporins only	38 (86.4%)	28 (80.0%)	
Second-generation cephalosporins	4 (9.1%)	6 (17.1%)	
Third-generation cephalosporins	39 (88.6%)	29 (82.9%)	
Cephalosporins combination	5 (11.4%)	7 (20.0%)	
Others	0	1 (2.9%)	
1 empiric antibiotic	38 (86.4%)	28 (80.0%)	
≥2 empiric antibiotics	6 (13.6%)	7 (20.0%)	
Antibiotics at home	9 (20.4%)	5 (14.3%)	
Prehospitalization antibiotics	13 (29.5%)	14 (40.0%)	
Glucocorticoids plus antibiotic therapy ^e^	40 (90.9%)	26 (74.3%)	* 0.048
Immune-boosting drugs	1 (2.3%)	4 (11.4%)	0.232

^d^ Empiric antibiotics: cephalosporin combination: a cephalosporin (cefoxitin sodium, ceftriaxone sodium, cefotaxime, cefaclor, and cefixime) plus either clindamycin, macrolide antibiotic (azithromycin), or glycopeptide (teicoplanin or vancomycin)—Other: clindamycin and fluoroquinolone (levofloxacin). ^e^ Glucocorticoids: dexamethasone sodium phosphate, prednisone acetate, and methylprednisolone sodium succinate. Abbreviations: DOTs, days of antibiotic therapy. * Denotes a significant difference between methicillin-resistant *S. aureus* and methicillin-susceptible *S. aureus* at *p* < 0.05.

**Table 3:** Comparison of the efficacy of glucocorticoid use between MSSA and MRSA.

	Length of Stay	*p* Value	Cure	Improvement	*p* Value
MRSA		0.273			<0.001 *
Antibiotics plus glucocorticoid therapy	6.92 ± 1.67		19	7	
Antibiotics use only	6.22 ± 1.56		4	5	
MSSA		0.305			0.024 *
Antibiotics plus glucocorticoid therapy	6.60 ± 1.82		31	9	
Antibiotics use only	12.00 ± 8.76		1	3	

* Denotes a significant difference between antibiotic plus glucocorticoid therapy and antibiotic use only for each item at *p* < 0.05.

## Data Availability

All the data generated or analyzed during this study are included in this article. Further enquiries can be directed to the corresponding author.
